# Epithelial-Mesenchymal Transition in Skin Cancers: A Review

**DOI:** 10.1155/2019/3851576

**Published:** 2019-12-16

**Authors:** Anastasia Hodorogea, Andreea Calinescu, Mihaela Antohe, Mihaela Balaban, Roxana Ioana Nedelcu, Gabriela Turcu, Daniela Adriana Ion, Ioana Anca Badarau, Catalin Mihai Popescu, Raluca Popescu, Cristiana Popp, Mirela Cioplea, Luciana Nichita, Ionela Hulea, Alice Brinzea

**Affiliations:** ^1^“Carol Davila” University of Medicine and Pharmacy, 050474 Bucharest, Romania; ^2^Colentina Clinical Hospital, 020125 Bucharest, Romania; ^3^Derma 360° Clinic, 011273 Bucharest, Romania; ^4^National Institute for Infectious Diseases Prof. Dr. Matei Balș, 021105 Bucharest, Romania

## Abstract

Epithelial-mesenchymal transition (EMT) is involved in physiologic processes such as embryogenesis and wound healing. A similar mechanism occurs in some tumors where cells leave the epithelial layer and gain mesenchymal particularities in order to easily migrate to other tissues. This process can explain the invasiveness and aggressiveness of these tumors which metastasize, by losing the epithelial phenotype (loss of E-cadherin, desmoplakin, and laminin-1) and acquiring mesenchymal markers (N-cadherin). Complex changes and interactions happen between the tumor cells and the microenvironment involving different pathways, transcription factors, altered expression of adhesion molecules, reorganization of cytoskeletal proteins, production of ECM-degrading enzymes, and changes in specific microRNAs. The purpose of this review is to determine particularities of the EMT process in the most common malignant cutaneous tumors (squamous cell carcinoma, basal cell carcinoma, and melanoma) which still have an increasingly high incidence. More studies are required on this topic in order to establish clear correlations. High costs related to skin cancer therapies in general as well as high impact on patients' quality of life demand finding new, reliable prognostic and therapeutic markers with significant public health impact.

## 1. Introduction

Epithelial-mesenchymal transition (EMT) is a complex biological process by which epithelial cells acquire special properties that make them more capable of undergoing embryogenesis and promoting normal wound healing. In contrast with these two physiologic aspects, EMT can also take place in the late carcinogenesis, promoting tumor progression and metastasis. During EMT, epithelial tumor cells leave their differentiated properties in order to obtain a mesenchymal-like phenotype, that makes them more invasive and more aggressive, allowing them to migrate into the surrounding tissues [1].

**Figure 1:**
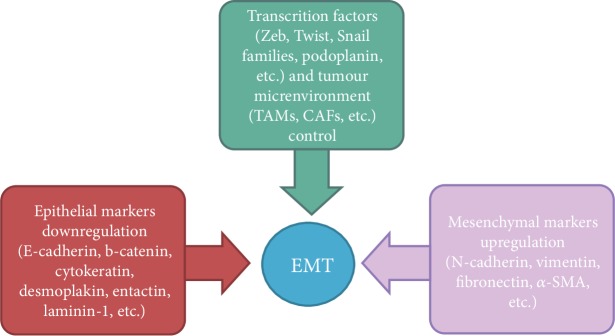
Epithelial-mesenchymal transition hallmarks.

The hallmarks of EMT *in vitro* and *in vivo* include the acquisition of a spindle-like/fibroblastic morphology, the upregulation of mesenchymal markers and extracellular matrix components, the downregulation of epithelial cell surface markers and cytoskeleton components, and the upregulation and/or nuclear translocation of specific transcription factors (i.e., Snail, Slug, Zeb1/2, and Twist1/2) [2] (Figure 1).

EMT implies losing cell-cell junctions and cell polarity. During this process, both gap and adherent junctions are lost. Cadherin-mediated adhesion is a dynamic process that is regulated by several signal transduction pathways. There is also evidence that cadherins are not only targets for signaling pathways that regulate adhesion but also may themselves send signals that regulate basic cellular processes, such as migration, proliferation, apoptosis, and cell differentiation [3, 4].

All these changes lead to the loss of basal membrane integrity. Moreover, there are cytoskeletal changes regarding the distribution of actin and the replacement of the cytokeratin filaments with vimentin [5, 6].

Single cells can invade lymphatic and hematogenous routes and induce distant metastasis. This phenomenon is facilitated by a decreased expression of E-cadherin, a subtype of cell adhesion molecule expressed by the epithelial cells. This protein is considered a key epithelial marker with tumor suppressor function that inhibits invasion and metastasis. A proof for this is a low transcription of its gene in various malignancies [7]. Moreover, other epithelial markers (cytokeratin, desmoplakin, entactin, and laminin-1) are lost and the cells acquire a mesenchymal phenotype through an increased expression of mesenchymal markers (neural cadherin (N-cadherin), vimentin, fibronectin, and smooth muscle actin alpha (*α*-SMA)) [1]. It has been shown that these cells with mesenchymal phenotype are often found in the invasion front of primary tumors being involved in invasion and metastasis processes [8].

The EMT process is controlled and enhanced by various transcription factors depending on the skin tumor (such as Zeb, Twist, Snail families, and podoplanin), expressed not only by cancer cells but also within the tumor microenvironment. The microenvironment, also known as the tumor stroma, is composed of tumor-associated macrophages (TAM), cancer-associated fibroblasts (CAFs), lymphocytes, and many other immune cells, that were proved to favor tumor progression and dissemination [9].

Snail and Zeb directly lower E-cadherin expression [10] while other factors, as Twist, have an indirect effect [11]. The maintenance of the epithelial cell polarity is provided by three protein complexes: Par, Crumbs, and Scribble, regulated by the EMT inductors [12]. During EMT, epithelial cell polarity is lost as a result of the Snail 1 suppressor action on Crumbs3 transcription and the loss of Par and Crumbs protein complexes at a junction level [13]. Zeb1 also has suppressive action on gene transcription of cellular polarity by inhibiting Crumbs3 and other genes [14]. TGF*β* plays an important role in the loss of cellular polarity in the EMT process, on the one hand, by expressing the Snail and Zeb genes and, on the other hand, by modifying the cytoskeletal architecture [15]. Snail and Zeb transcription factors promote invasion by the expression of matrix metalloproteinases (MMPs) that play a role in destroying the basement membrane. Moreover, MMP3 stimulates the production of reactive oxygen species, thus inducing Snail1 expression and ultimately triggering EMT [16]. Transcription factors confer malignant traits, such as motility, invasiveness, and resistance to apoptosis on neoplastic cells [10, 17–21].

The EMT process can be reversible as the mesenchymal cells become epithelial cells when they reach the secondary sites. This process known as mesenchymal-epithelial transition (MET) facilitates the formation of metastasis [1].

Another process involved in cancer metastasis is collective cancer invasion, in which a group of neoplastic cells, with preserved cell-cell adhesion, move away from the primary tumor. In this case, only a few cells suffer EMT, in order to head the entire group [22–24].

Being so evident of the implication of EMT in cancer progression, in aggressiveness, the aim of this review is to assess different aspects of EMT in the most common malignant skin tumors (squamous cell carcinoma (SCC), basal cell carcinoma (BCC), and melanoma) whose incidence is alarmingly increasing but still with limited therapeutic targets.

## 2. EMT in Cutaneous SCC

Cutaneous squamous cell carcinoma (cSCC) is easily treated and the cure rate is high, but there are cases where metastasis can occur. An accurate clinical exam correlated with a histological and immunohistochemical investigation can establish the biomarkers involved in the development and evolution of this malignancy and reveal the appropriate treatment for each patient.

EMT in the setting of cSCC is a process far from being completely understood. As within other skin cancers, the phenomenon has been explained through two important aspects in the cSCC metastasis process: on the one hand, the loss of the expression of epithelial markers in order to invade and disseminate from the primary tumor, on the other hand, the need to revert to an epithelial identity in order to form metastases to distant sites [8]. This hypothesis could have major implications for the management of cSCC, questioning whether therapeutic agents that inhibit EMT [25] or therapeutic agents that inhibit the reversion of EMT [26] would be more appropriate to be used as a treatment. Animal model studies are expected to play major roles in assessing different management strategies designed for skin carcinomas [27].

Often involved in the transformation into a mesenchymal-type phenotype is the acquisition of vimentin and the loss of cell-cell attachment molecules E-cadherin and beta-catenin [26, 28, 29]. Adhesion proteins such as E-cadherin are essential in maintaining cellular integrity. Results from studies demonstrating a reduction in the expression of membranous E-cadherin on cSCC cells, when compared to precursor cSCC lesions and normal skin, indicate EMT as an important process in cSCC progression [30]. Membranous E-cadherin is bound to beta-catenin, which is released when the former is downregulated and can translocate to the nucleus, being able to activate genes involved in proliferation and invasive growth [31]. Membranous E-cadherin expression appears to be correlated with the degree of tumor differentiation, with upregulation in well-differentiated SCCs and attenuated or missing staining in poorly differentiated tumors [24, 32]. Instead, poorly differentiated SCCs seem to have a high cytoplasmic expression of E-cadherin [24]. This translocation from the membrane to the intracytoplasmic region is regarded by many as functional loss of this adhesion molecule, attenuating cellular integrity and thus, promoting malignant transformation and metastasis in the setting of EMT [33–36]. Further evidence that point to EMT as a tumor progression indicator in cSCC is delivered by studies showing a decrease of membranous E-cadherin in corresponding lymph node metastases when compared to primary cSCCs [24, 37]. The membranous downregulation is again accompanied by increased cytoplasmic staining [24, 34].

**Figure 2:**
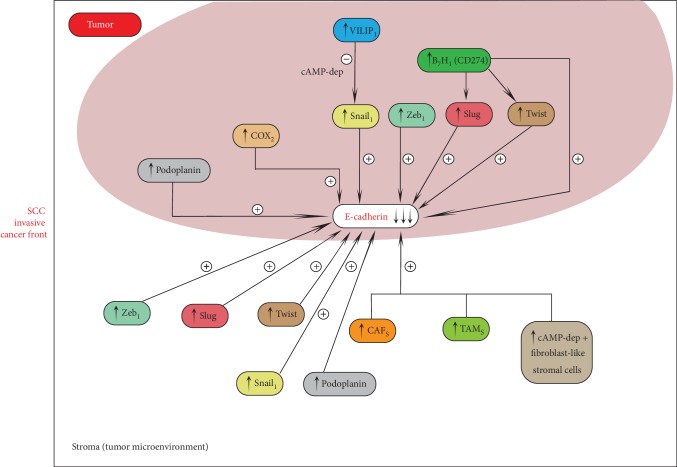
SCC cancer invasive front.

We will shortly review herein the most important factors reported to influence EMT in cSCC (Figure 2).

Aberrant expression of several transcriptional repressors including Zeb1, Slug, and Twist induces E-cadherin downregulation at the invasive cancer front [38, 39]. These EMT-related proteins can also be expressed by cells from the tumor stroma, such as cancer-associated fibroblasts (CAFs). Activated CAFs are believed to promote tumor progression and decrease patient survival [40–42]. Sasaki et al. analyzed the expression of proteins related to EMT and CAF in different skin cancers, showing that the microenvironment at the tumor invasive front shows different specific expression patterns in cutaneous BCC, SCC, and MM [9]. High expression levels of podoplanin, PDGFR*β*, CD10, S100A4, *α*-SMA, Zeb1, Slug, and Twist were obtained in the group of cSCCs. The result could represent a useful panel of biomarkers in order to assess skin cancer invasiveness [9].

The contribution of the tumor microenvironment to tumor invasiveness and metastasis is also portrayed by the role of tumor-associated macrophages (TAM). They are shown to induce Snail promoter activity and EMT in MCF-7 breast cancer cells via TNF-*α* [43] and have been found in higher numbers in cSCC and Bowen's disease (BD) when compared to precancerous lesions [44]. In addition, the cell surface zinc-dependent metalloprotease CD10, expressed in the peritumor fibroblast-like stromal cells of the invasion front of various malignancies [45–48], appears to be increased in cSCC compared to precancerous lesions.

Podoplanin, a mucin-type transmembrane glycoprotein, mediating cellular contractile properties and cytoskeletal reorganization, is upregulated at the leading edge of the tumor in metastatic and poorly differentiated cSCC [24]. Studies comparing primary nonmetastatic cSCC, primary metastatic cSCC, and their corresponding lymphatic metastases demonstrate podoplanin, Twist, Zeb 1, vimentin, and beta-catenin overexpression in metastatic cSCC, with Twist ectopic expression inducing Zeb1, vimentin, and podoplanin expression and also E-cadherin delocalization, resulting in scattered migration pattern *in vitro* [33]. However, EMT marker expression was decreased in metastases compared to the corresponding primary tumors [33]. Overexpression of podoplanin represented a statistically independent prognostic factor for disease-free survival in other studies [24].

PGE2–EP2 signaling pathway is also believed to play an important role in EMT mediation by contributing to E-cadherin downregulation during ultraviolet- (UV-) induced cSCC progression [35]. Cyclooxygenase 2 (COX-2) overexpression in cSCC and precursor lesions was reported in various studies [49–52], and it seems to occur together with inactivation of E-cadherin [53].

B7-H1 (CD274), a T-cell coinhibitory molecule, often expressed in human carcinoma cells, believed to be implicated in the immune escape process also appears to favor EMT. Murine models studied by Cao et al. investigating its expression in a murine methylcholanthrene- (MCA-) induced model of SCC revealed that upregulation of B7-H1 in skin epithelial cells downregulates E-cadherin and upregulates Slug and Twist, promoting EMT [54].

Visinin-like protein 1 (VILIP-1), a neuronal calcium sensor protein, putative tumor migration suppressor gene, modulating cyclic nucleotide levels and inducing cell differentiation, appears to be involved in the process of EMT in cSCC. Studies on SCC mouse model cells by Schönrath et al. found that VILIP-1 suppresses the expression of the EMT-related transcriptional repressor Snail1 in a cAMP-dependent manner [55]. The induction of Snail is inhibited by elevated cAMP levels [55]. Mahloogi et al. used also murine cSCC cells and reported that ectopic expression of VILIP-1 in high-grade SCC lines that did not express VILIP-1 increased cAMP levels, decreased MMP9 and RhoA activity, reducing the invasiveness of the SCC cells [56]. Gonzalez Guerrico et al. suggested that VILIP-1 reduces cell adhesiveness, migration, and invasiveness thorough decreasing fibronectin-specific integrin [57].

In spite of the majority of studies gathering overwhelming proof of EMT implication in cSCC progression, conflicting results by few studies indicate high, predominantly membranous expression of E-cadherin in primary cSCC and cSCC skin metastases. These results dispute the implication of EMT in SCC progression favoring the hypothesis of collective cancer invasion [24]. In this process, adherent cell groups are believed to detach from the primary tumor, favoring malignant transformation and metastasis. Therefore, E-cadherin upregulation is essential to maintain cellular integrity. However, even in this setting, a small number of cells at the leading edge of the adherent cell complex presumably undergo EMT, in order to provide guidance [24].

### 2.1. Discussions

Different authors have analyzed the role of EMT in cutaneous squamous cell carcinogenesis, using human and animal models, highlighting the expression and activity of epithelial and mesenchymal markers, transcription regulatory factors, and relevant intra- and extracellular pathways.

We identified studies that have investigated EMT contribution to skin SCC mechanisms, current topics of high concern for medical and scientific research. Thus, there is evidence of EMT involvement in actinic keratosis progression into invasive cSCC [58], EMT upregulation in the invasive cSCCs compared to normal skin and with cSCCs *in situ* [30, 59], a particular immunohistochemical pattern of EMT-related protein expression in SCCs [9], and EMT reversion at distant metastasis sites [8, 26].

New insights into the mechanisms of metastasis in SCCs may reveal the distinct contribution of collective cancer invasion and single-cell invasion pathways, in order to optimize the treatment strategy of these patients.

## 3. EMT in Basal Cell Carcinoma

**Figure 3:**
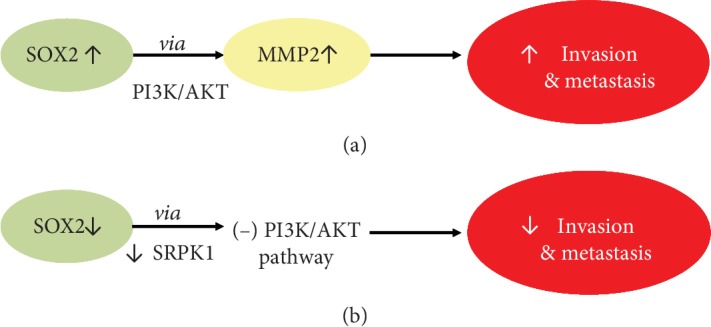
SOX2 pathway in cancer cells (a) vs. BCC cells (b).

EMT is also a critical regulator in the progression of cancer metastasis in BCC through SOX2 expression that regulates the EMT processes and proliferation of BCC cells. Some studies showed that overexpression of SOX2 promotes human cancer cell proliferation mainly through promoting migration and invasion via PI3K/AKT by increasing MMP2 expression. In BCC, downregulation of SOX2 leads to low expression of SRPK1 which inhibits the PI3K/AKT signaling pathway decreasing migration and invasion. These data suggest that SRPK1 may be a direct target of SOX2-induced EMT processes in BCC cells as reduced expression of SOX2 may lead to suppression of BCC metastasis (Figure 3). This could be an explanation of why BCCs are usually less aggressive. However, in the very few cases when BCC becomes invasive, it was reported that activation of the PI3K/AKT signaling pathway may abrogate the effects of SOX2 knockdown on BCC cell migration and invasion [60].

Papanikolaou et al. found in all 100 cases of human BCC in their study that ILK (Integrin-Linked Kinase) was overexpressed and it was strongly correlated with tumor invasion and also with EMT features (loss of E-cadherin, Snail, nuclear *β*-catenin, and *α*-SMA expression) [61].

Majima et al. showed that tumor cells were positive for Twist1 at the invasive front of the primary tumor, whereas the tumor cells centrally were negative for Twist1.

In nonmetastatic BCC (nodular BCC), tumor cells were Twist1 negative. Double immunofluorescence stains for E-cadherin and N-cadherin showed that E-cadherin was prominently expressed in nodular BCC, whereas this epithelial marker was markedly decreased in the tumor cells of metastatic BCC. For N-cadherin, the tumor cells were negative in nodular BCC and markedly positive in tumor cells at the invasive front of metastatic BCC. Twist1 and N-cadherin were highly expressed in metastatic tumor cells, and E-cadherin expression was markedly decreased in the metastatic tumors. Twist1 is capable of promoting EMT, contributing to aggressive invasion and multiple organ metastases. The expression levels of Snail, a direct transcriptional repressor of E-cadherin, the other transcription factor have been shown to correlate with the depth of tumor invasion in BCC [62].

Tumors of epithelial origin can express transcription factors Snail and Twist1, or the cell adhesion molecule N-cadherin as a mesenchymal marker.

### 3.1. Discussion

The association of SOX2 expression with the progression of other several human cancer cells has been reported [63–65] but the role of SOX2 in these cancers remains controversial [66] as Yang et al. [67] reported that SOX2 promotes the migration and invasion of laryngeal cancer cells by induction of MMP2 via the PI3K/AKT/mechanistic target of the rapamycin pathway, while Yoon et al. [68] indicated that overexpression of SOX2 is associated with better overall survival in squamous cell lung cancer patients treated with adjuvant radiotherapy.

The SRPK1/PI3K/AKT pathway may be involved in the role of SOX2 in the migration and invasion of BCC cells, and this is why SOX2 may be a novel potential therapeutic target for BCC [60]. SRPK1 is a protein that is dysregulated also in other types of cancer, and this is why SRPK1 inhibition is considered a potential therapeutic target in prostate cancer [69]. A study indicated that SRPK1 has a critical role in the EMT process of human glioblastoma too [70]. It was demonstrated that SRPK1 functions as an oncogene by promoting the activation of PI3K/AKT signaling [71], a pathway involved in the development and progression of human cancer, very well described in lung cancer [72–75].

Meanwhile, knockdown of SOX2 inhibits BCC cell proliferation by upregulating E-cadherin expression and also by lowering vimentin and fibronectin and also by downregulation of the SRPK1-induced EMT signaling pathway [60].

The immunofluorescence assay also confirmed the effects of SOX2 knockdown and overexpression on the epithelial and mesenchymal marker expression levels in BCC cells. SRPK1 overexpression canceled the SOX2 knockdown-inhibited EMT processes of BCC cells. These data suggest that SRPK1 is a direct target of SOX2-induced EMT processes in BCC cells [60].

Aberrant expression of E-cadherin, nuclear beta-catenin, and alpha-SMA correlated with BCC tumor invasion.

In BCC expression, levels of Snail were correlated with the depth of tumor invasion, whereas in cSCC, there is no significant expression of Snail.

## 4. EMT in Melanoma

Melanocytes are cells derived from neuroectoderm, and during their migration to the epidermis, in the fetal period, they undergo numerous changes similar to EMT-MET ones, thus preserving some particularities. Because of their different origin than the other epidermal cells, melanoma cells were observed to experience a distinct EMT development than other tumor cells derived from the epidermis.

Normally, keratinocytes keep melanocytes from leaving the epidermis through E-cadherin, which is a cell-cell adhesion molecule not present between melanocytes [76, 77]. This molecule is no longer expressed when melanoma cells leave the epidermal layer, suggesting that they lose their epidermal properties, acquiring new specific mesenchymal changes which promote melanoma's invasiveness and progression [78–80]. In a study of Diana et al. which included nevi and dysplastic nevi, the other cell-cell adhesion molecule involved in the EMT, N-cadherin, was present only in the dermal component, being absent in the epidermal or junctional areas, highlighting the idea that its positivity shows a potential malignant transformation of nevi [81].

In melanomas, it has been observed that when switching from radial growth phase (RGP) to vertical growth phase (VGP), there is downregulation of E-cadherin, P-cadherin, and H-cadherin expression, explaining the loss of keratinocyte control over melanoma cells. This is how melanoma cells are gaining properties to evade the epidermis [82–84]. Melanoma is believed to progress characteristically by alternating between proliferative and invasive states, the presence of both types of melanoma cell phenotypes in the same tumor being the main argument [85, 86].

**Figure 4:**
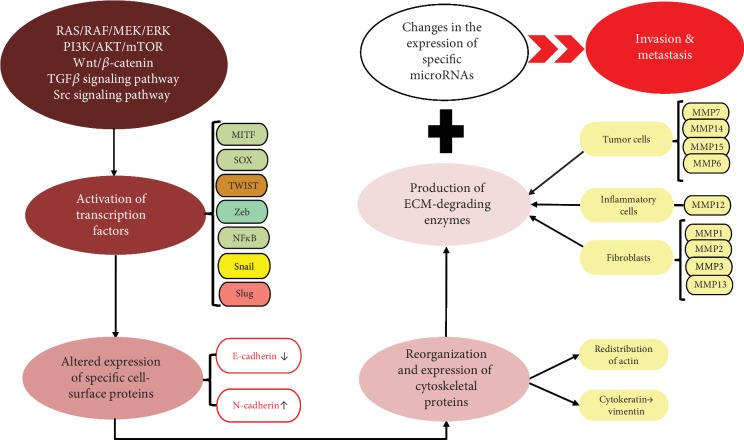
Progression, migration, and invasion pathways in melanoma EMT.

Several pathways are incriminated in the EMT: RAS/RAF/MEK/ERK, PI3K/AKT/mTOR, Wnt/*β*-catenin, and Transforming Growth Factor *β* (TGF*β*), Src—and subsequently their effectors, transcription factors—such as microphthalmia-associated transcription factor (MITF), SOX Family, Snail, Slug, Twist, Zeb, and NF*κ*B [2]. An illustrative overview of the progression, migration, and invasion pathways and key points in the EMT of melanoma is described in Figure 4.

Caramel et al. proved in their study that there is a switch in EMT transcription factors between Snail2 and Zeb2 which are found in normal melanocytes and Zeb1 and Twist1 seen in melanoma, changes acquired through the MEK/ERK oncogenic pathway. These modifications were seen to be happening gradually starting from the superficial areas of the melanomas, respectively, from the cortical area of the lymph node metastases (Zeb2/Snail2 positive and Zeb1/Twist1 negative) until the deeper parts of the tumor and the medullar part of the metastatic lymph nodes (Zeb2/Snail2 negative and Zeb1/Twist1 positive) [87].

MITF, which is a transcription factor involved in melanocyte development and differentiation, was found to be regulated by Zeb2. The switch between Zeb2 and Zeb1 inside the melanoma was observed to be correlated with reduced expression of MITF and consequently with tumor progression [87, 88].

The interaction between beta-catenin, lymphoid enhancer-binding factor 1 (LEF1), and transcription factor 4 (TCF4) was studied in order to see its effects on melanoma. It was observed that the increase in TCF4 and decrease in LEF1 was associated with an invasive transformation of melanoma, in contrast with epithelial tumors where the upregulation of beta-catenin interaction factor LEF1 was seen to promote EMT [89, 90]. The Wnt signaling pathway controls the complex beta-catenin/LEF1 resulting in the regulation of MITF [91].

Another pathway believed to be involved in the EMT of melanoma cells is the oncogenic Notch pathway; however, therapy with Notch inhibitors was not effective on metastatic melanoma until now [92]. The Notch1 signaling pathway increases N-cadherin expression in mesenchymal melanoma cells. As a consequence, the malignant melanocytes acquire a more aggressive phenotype by increasing their invasiveness. Diana et al. observed that while a high presence of Notch1 or N-cadherin alone in the melanoma or the metastases did not bring any significant correlations with overall survival of the patients. The high expression of both Notch1 and N-cadherin in the same lesions correlated with poor prognosis. The authors suggest that this coexpression should be taken into account as a prognostic factor for melanoma patients [81].

In contrast to Notch1, Notch4 was seen to induce opposite changes in melanoma cells, reexpressing epithelial markers (MET-like changes as explained in the introduction). By increasing E-cadherin expression and decreasing Snail2, Twist, vimentin, and MMP2 expression, Notch4 reverts the progressive and invasive pattern of EMT and succeeds in tumor suppression. This is believed to be a reason for the yet unsuccessful therapy with Notch inhibitors [93].

Podoplanin, introduced in the paragraph about SCC, is another researched molecule believed to play different roles in the EMT of some cancers, by losing epithelial-specific markers such as E-cadherin and gaining mesenchymal markers, among which are N-cadherin and fibronectin [94]. It was found to be absent in normal melanocytes and fibroblasts, but present in almost 69% of the melanoma patients studied by Kan et al., yet without any significant correlation with tumor progression or overall survival. However, they observed a worse prognosis and a higher risk for metastases for melanomas with podoplanin positive tumor-associated fibroblasts, suggesting its role as a potential prognostic marker and therapeutic target [95].

As far as immune cells and EMT are concerned, melanoma cells exhibit an interesting particularity. When the change in phenotype is acquired, epithelial to mesenchymal, tumor antigens modify, thus escaping immune surveillance. This is why it is important to target antigens which are common to both epithelial and mesenchymal phenotypes when trying to develop immune therapy [96]. NK cells were seen to promote the change from the proliferative to the invasive state [97]. TAM induce EMT through TGF*β* by secreting IL6, IL1, TNF*α*, and MMPs [98].

Moreover, in order for the EMT to take place, melanoma cells are required to pass through the basement membrane and ECM (extracellular matrix). Specific molecules which have the role of degrading proteins are called matrix metalloproteinases and are released in the tumor environment by tumor cells (MMP7, 14, 15, and 16), inflammatory cells (MMP12), and fibroblasts (MMP1, 2, 3, and 13). When their enzymatic activity surpasses their inhibitors, called tissular inhibitors of matrix metalloproteinase (TIMPs), they favor tumor cells acquire invasiveness and aggressiveness facilitating EMT. As far as TIMPs are concerned, it was observed that an increase in their expression is correlated with a decrease in melanoma's invasiveness [99–102].

60% of melanomas have V600E BRAF mutation. Vemurafenib is a competitive kinase inhibitor with activity against BRAF kinase with V600E that interrupts the B-Raf/MEK step on the B-Raf/MEK/ERK pathway (Figure 4). Patients treated with Vemurafenib had a median overall survival of 14 to 16 months, which significantly improved comparing to the classical treatments, that induced a median survival period of 6 to 10 months [103]. Frequently, after an initial response, the disease progresses due to MEK reactivation [104].

It has been suggested for the melanoma patients who are resistant to Vemurafenib to associate an inhibitor of the EMT. This approach was proposed after noticing cell migration and phenotype switching in these drug-resistant patients while only suppressing the oncogenic signaling pathway BRAF is not sufficient. The targeted molecule was TGF*β*, and its inhibition associated with Vemurafenib was seen to defeat the resistance [105]. Other suggestions of future therapies involving EMT were changing the phenotype of melanoma cells to a targetable one or by shifting the cadherin switch [106, 107].

## 5. Discussion

The differences between melanoma and keratinocyte carcinomas highlight the idea that EMT is a polymorphic and distinct phenomenon with element characteristic to each type of tumor in order to best integrate the cells in the microenvironment [108].

It is assumed that melanoma can metastasize faster than other malignant skin tumors, being more aggressive because normal melanocytes possess from the start elements which contribute to EMT, such as vimentin or some transcription factors (SNAIL2, ZEB2) [109, 110].

Melanoma is a sneaky tumor which has a dynamic character changing back and forth between phenotypes. Due to the unique diversity and plasticity of melanoma cells, it is hard to treat this skin tumor and to foresee its evolution and prognosis.

## 6. Conclusion

Further studies are needed to assess the onset time of EMT during the process of cutaneous carcinogenesis. Summarizing, the EMT process may influence each stage of skin carcinogenesis, from premalignant changes to distant macrometastatic tumorigenesis. The rationale of inhibiting EMT or inhibiting the reversion of EMT during therapeutic management should also be clarified.

Although the loss of E-cadherin is a critical step in EMT, alone it is not necessarily sufficient to drive EMT.

In this paper, we have focused on the involvement of epithelial-mesenchymal transition in skin cancer mechanisms. We discussed the role of EMT events in cutaneous melanoma, basal cell carcinoma, and squamous cell carcinoma. The results are suggestive rather than conclusive regarding the pathogenic contribution of EMT in different skin neoplasia pathways. The relative paucity of the scientific literature on this topic, the quantitative and qualitative limitations of some studies lead to the need for further insights in order to decipher: EMT contribution to the natural history of cutaneous malignancies, the potential use of EMT markers for an optimized diagnostic staging, and the relevance of the therapeutic modulation of EMT steps.
